# Bilateral carotid cavernous fistula after trauma: a case report and literature review

**DOI:** 10.1186/s41016-021-00265-x

**Published:** 2021-11-04

**Authors:** Jingshan Liang, Xiaoxiao Xie, Yong Sun, Xiuli Wei, Aimin Li

**Affiliations:** 1 Department of Neurosurgery, The Affiliated Lianyungang Hospital of Xuzhou Medical University/The First People’s Hospital of Lianyungang, No. 182, Tongguan Road, Lianyungang, 222000 Jiangsu China; 2 Department of Neurointervention, The Affiliated Lianyungang Hospital of Xuzhou Medical University/The First People’s Hospital of Lianyungang, Lianyungang, Jiangsu China; 3 Department of Internal Nursing, Lianyungang Higher Vocational Technical School of Chinese Medicine, Lianyungang, China

**Keywords:** Carotid cavernous fistula, Bilateral, Trauma, Endovascular embolization

## Abstract

**Background:**

Carotid cavernous fistula is a rare complication that is typically associated with head trauma and skull base fractures. The traumatic bilateral carotid cavernous fistula are significantly rarer.

**Case presentations:**

We report a case of a 61-year-old man presenting with unilateral exophthalmos, swollen eyelids, conjunctival congestion, and edema etiologically associated with severe trauma. Thereafter, the patient demonstrated symptoms of contralateral oculomotor nerve injury caused by skull base fracture, such as ptosis of eyelid, dilated pupils, and eye movement disorder, and was diagnosed with bilateral carotid cavernous fistula.

**Conclusions:**

The patient recovered after undergoing endovascular embolization of bilateral cavernous sinus fistulas. The patient demonstrated the classic symptoms of an extremely rare condition known as bilateral carotid cavernous fistula, in only one eye. Reporting and analyzing this case will help us elucidate the underlying mechanisms of this disease.

## Background

Carotid cavernous fistula (CCF) is an abnormal arteriovenous communication formed between carotid artery and its branches due to the influence of external factors such as trauma, or spontaneous and cavernous sinus, resulting in a series of clinical symptoms and signs associated with the blood stealing and increased venous pressure. The disease is typically caused by head trauma and skull base fracture and has an incidence rate of 1-2.5% in cases of head trauma [5, 7]. Traumatic CCF cases are characteristically unilateral, and those of bilateral traumatic carotid cavernous fistula (TCCF) are extremely rare. The clinical symptoms of CCF are associated with the direction of venous drainage. The most common etiology of ocular venous drainage is characterized by the presence of the “triple syndromes” typically associated with carotid cavernous fistula in a majority of the patients with intracranial vascular murmur, pulsatile exophthalmos, bulbar conjunctival congestion, or edema. Ocular venous drainage will have “triple syndrome” manifested by intracranial vascular murmur, pulsating eyeball, conjunctival hyperemia, or edema, usually associated with carotid cavernous fistula [14]. We believe that this case of bilateral CCF with pertinent symptoms in only one eye is very rare and combined with contralateral oculomotor nerve injury, it may be easily misdiagnosed and missed and, therefore, deserves our attention.

## Case presentation

### History and examination

**Fig. 1:**
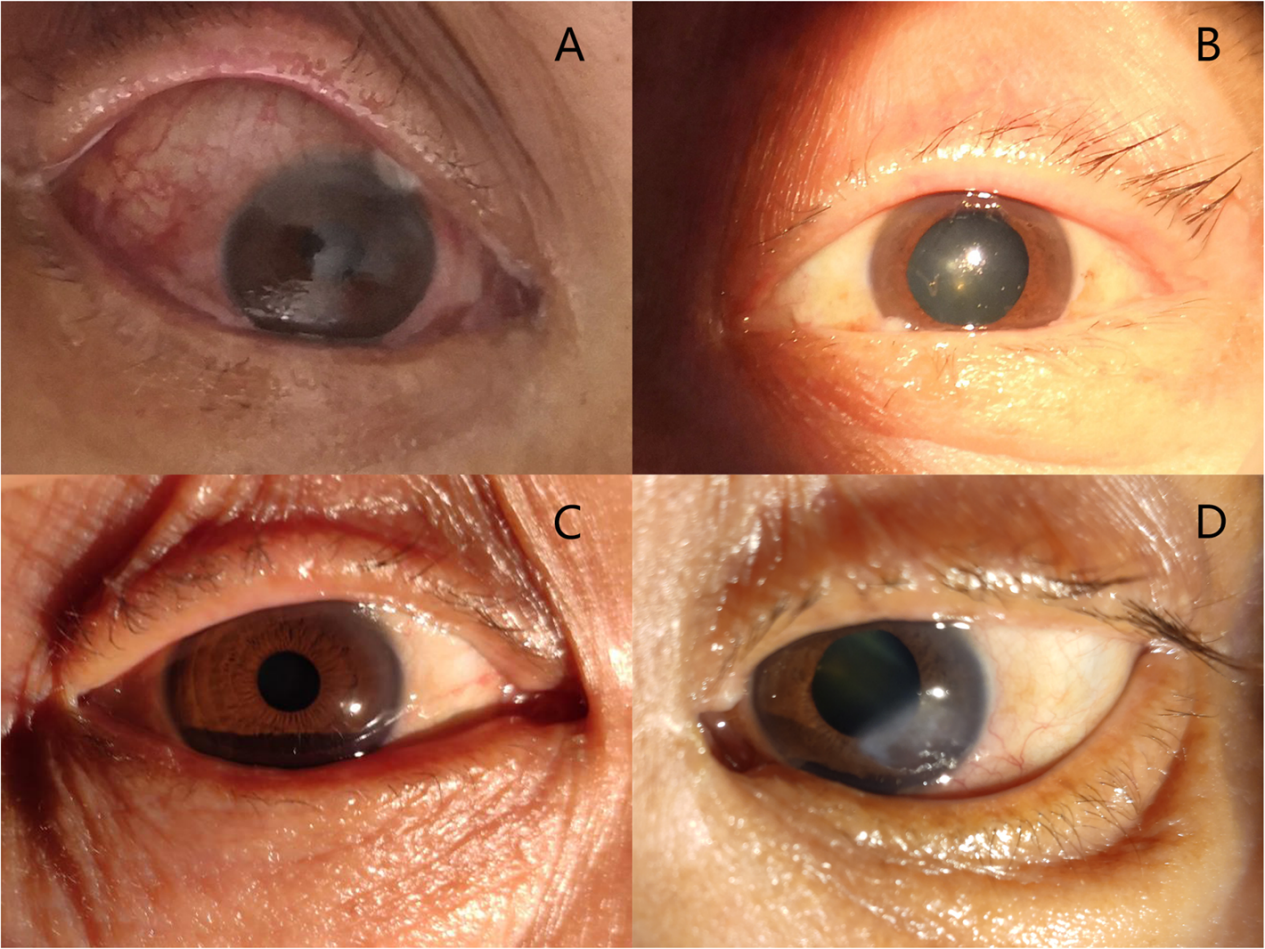
**A** Preoperative patient with right eye protrusion; eye movement limitation, conjunctival congestion, and edema are obvious. **B** The left eyelid is drooping, the pupil is dilated, the eyeball is inactive, and the light reflection disappears, but there is a light sensation. **C** One month after the surgery, the patient’s right eye protrusion, conjunctival congestion, and edema show significant relief, and the eye movement returns to normal after 6 months. **D** There is no significant change in the left eye

A 61-year-old man accidentally hurt his head while cutting trees 3 months ago, followed by loss of consciousness and bleeding in the bilateral nasal and external auditory canal. He scored 7 on the Glasgow coma scale (GCS). CT examination revealed extensive subarachnoid hemorrhage, skull base fracture, intracranial pneumatosis, and double lung contusion. The patient was treated conservatively after admission. Physical examination showed 5-mm dilation in the left pupil, along with an absence of pupillary light reflex to direct and indirect light sources. The right pupil was dilated by 3 mm, and pupillary light reflex was slower than normal. Furthermore, bilateral eye movements could not be examined, and the bilateral eyelid and conjunctival edema were not obvious. On the 13th day of hospitalization, there was a marked improvement in the patient’s consciousness; he could automatically blink and was able to easily follow simple instructions. Consequently, we noted a swelling in the patient’s right eyelid, along with conjunctival congestion, edema, and exophthalmos. The eyes seemed to pulsate on touch and auscultation revealed vascular murmur. The patient presented with drooping of the left eyelid, dilation of the pupils, and loss of direct and indirect pupillary light reflex. Although he was able to perceive light, and there were no edema or congestion present on the conjunctiva, he was unable to move his eyeball (Fig. 1A, B).

**Fig. 2:**
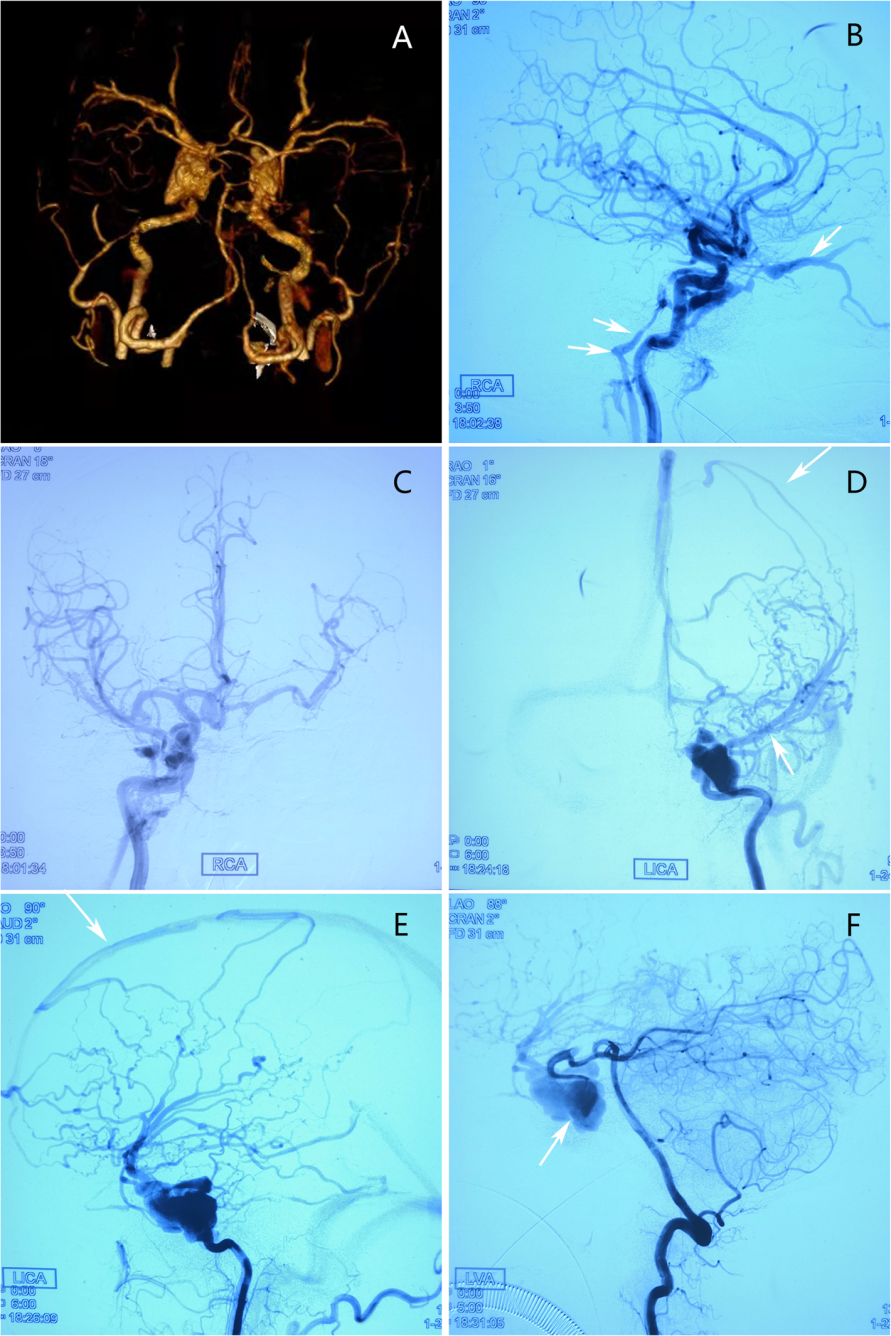
**A** CTA shows irregular tortuous dilatation of bilateral carotid cavernous sinus segment. **B** Right carotid angiography shows that the arterial blood flows through the cavernous sinus into the right superior ophthalmic vein (white arrow) and inferior petrosal sinus (double white arrow) and causes it to expand. **C** Right carotid angiography demonstrates that the anterior communicating artery supplies blood to the left cerebral hemisphere with good compensation. **D** Left carotid angiography demonstrating that the cavernous sinus fistula is relatively large, the distal artery blood flow has not yet developed, and the blood flow through the fistula is drained into the vein (white arrows), showing “stolen flow” phenomenon. **E** The left carotid angiography shows significant cortical venous reflux (white arrow). **F** Left vertebral artery angiography shows reverse flow of blood to cavernous sinus fistula via left posterior communication (white arrow)

Symptoms of his right eye were obvious and those of his left oculomotor nerve palsy appeared on admission. Therefore, we suspected that the patient had a CCF on the right side and a left oculomotor nerve injury associated with skull base fracture. Subsequently, we performed a CTA examination on the patient’s head and surprisingly observed that the contrast agent had extravasated from the cavernous segment of the bilateral internal carotid artery. Three-dimensional imaging revealed that the bilateral internal carotid arteries were swollen and irregular (Fig. 2A); therefore, we suspected that the patient may have a bilateral CCF. Further DSA examination showed that there was a crack on the medial side of the cavernous segment of the bilateral internal carotid artery and that the blood from the right internal carotid artery had flown into the cavernous sinus, and subsequently, flown back into the right superior ocular vein and the inferior petrosal sinus (Fig. 2B). Simultaneously, we also noted that the anterior communicating artery supplies blood to the left cerebral hemisphere (Fig. 2C). The flow-rate of the left fistula was relatively large, and the blood flowing from the internal carotid artery could not enter the distal end through the fistula and instead flowed into the venous system via the expanded cavernous sinus (Fig. 2D), and obvious cortical venous reflux (Fig. 2E). Additionally, angiography of the vertebral artery can be used to visualize the countercurrent blood flow to the cavernous sinus fistula via the left posterior communication artery (Fig. 2F). Considering the large amount of contrast agent that enters the cavernous sinus through the left carotid artery to form a large mass, which is represented as a dense shadow, it is difficult to determine the location of the fistula. The fistula was located using vertebral artery angiography.

### Operation

**Fig. 3:**
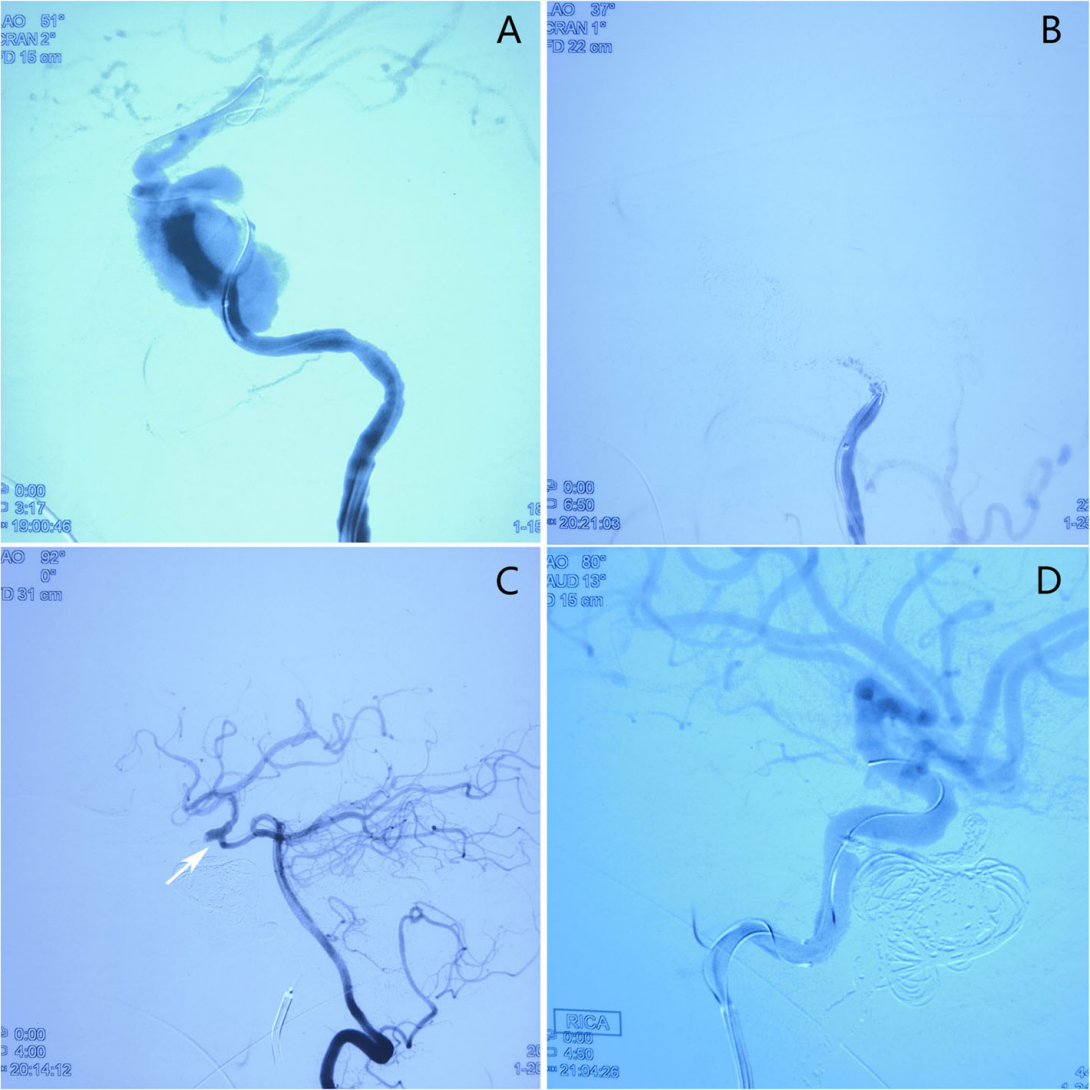
**A** Catheter is inserted through the left femoral artery, and the balloon is placed at the fistula of the carotid cavernous sinus, showing that the balloon is completely herniated into the fistula. **B** Left carotid artery angiography shows the occlusion of the left internal carotid artery and that the fistula is completely closed. **C** Left vertebral artery angiography shows no countercurrent blood flow (white arrow) through the left posterior communicating artery and cavernous sinus fistula is completely blocked. **D** Right carotid artery angiography shows that the drainage veins of right superior ophthalmic vein and inferior petrosal sinus disappears after embolization of fistula with microcoil and Onyx glue

The patient underwent bilateral femoral artery puncture under general anesthesia. The 6F sheath was placed in the right femoral artery and the 5F sheath was placed in the left femoral artery. Through sheath intubation, facilitated by total cerebral angiography, the guide catheter was led into the left internal carotid artery. The 4 × 20 mm balloon was placed at the fistula and filled. The balloon was completely herniated into the fistula, highlighting the huge size of the fistula (Fig. 3A). Therefore, we introduced coils to embolize the cavernous sinus and the fistula including both the distal and proximal ends, and the internal carotid artery, following which we performed angiography of the left common carotid and vertebral arteries which showed the occlusion of the left internal carotid artery and the disappearance of the cavernous sinus fistula (Fig. 3B, C). The guide catheter was subsequently introduced into the right internal carotid artery, and the microcatheter was introduced into the fistula, which was embolized with coil spring in combination with ONYX glue. Angiography revealed the disappearance of the right internal carotid cavernous fistula (Fig. 3D).

### Postoperative course

Studies [6, 8] have previously reported that patients with CCF recover after a period of intravascular embolization. Although the ocular symptoms, intracranial vascular murmurs, etc., recovered faster, the neurological paralysis took longer to completely recover. The symptoms of the patient’s right eye significantly improved after 1 month (Fig. 1C) and eye movement returned to normal after 6 months, and there was mild movement disorders at 7 days and recover well at 6 months after the operation; however, those of the left oculomotor nerve injury and vision did not demonstrate adequate improvement (Fig. 1D). It is of paramount importance to regularly follow up and monitor the patient’s condition, to successfully evaluate long-term prognosis.

## Discussion

Pathophysiological mechanisms, hemodynamics, and classification of bilateral CCF are more complicated than unilateral CCF. In 1985, Barrow et al. [1] comprehensively classified TCCF into four types: A, B, C, and D. This classification method focused on the vessels associated with the pathophysiology of CCF and is preferred by us.

CCF clinical manifestations are associated with the structure of cavernous sinus (CS) and draining vein. When the pressure in the CS is too high, the nerves are often forced to have clinical manifestations such as blepharoptosis, eye movement disorder, and diplopia. Upon damage to the cavernous segment of the internal carotid artery and branches of ICA or ECA, arterial blood flows directly into CS through the fistula. Increased pressure in CS leads to ocular vein dilation, followed by a series of ocular symptoms such as conjunctival edema, hyperemia, and exophthalmos. Additionally, bilateral CCF does not necessarily produce the same clinical manifestations due to differences in hernia size and hemodynamics [16]. In this case, the patient’s right internal carotid artery blood flows through the cavernous sinus and flows back into the right superior ocular vein and inferior petrosal sinus. Therefore, symptoms typically associated with the CCF triad appears. However, the left eye only shows the symptoms of oculomotor nerve injury etiologically associated with the blood flow from the left internal carotid artery through the ruptured cavernous sinus fistula, such as the cortical vein, without flowing directly into the ophthalmic vein. Additionally, differences in venous drainage directions and blood flow between arteriovenous shunts produce different clinical manifestation [2, 11, 15]. Another mechanism of traumatic CCF that leads to clinical symptoms is the phenomenon known as “blood theft,” which occurs when the arterial blood flows back to the vein through an abnormal route, which inevitably causes a decrease in blood perfusion in the posterior regions of the fistula. Severe “blood theft” may even lead to adverse events such as cerebral infarction in patients. Left carotid angiography can be used to note that the arterial blood flow at the distal end of the fistula is not visualized, rather flows into the vein, resulting in “blood theft.” Right carotid angiography shows that the anterior communicating artery adequately supplies blood to the left cerebral hemisphere, which suggests that circle of Willis is functioning normally, avoiding the occurrence of adverse events.

CTA is the first choice to diagnose CCF [4, 10], and DSA remains the gold standard to diagnose CCF [9, 13]. In the absence of the classic triad in the CCF patient, the condition can be easily missed and misdiagnosed, suggesting that it may be necessary to conduct routine head CTA examination in clinical settings for patients presenting with craniocerebral trauma, particularly for those with skull base fractures. It needs to be differentiated from intraorbital meningiomas, intraorbital aneurysms, and cavernous sinus thrombosis. In addition, pulsating exophthalmos and intracranial vascular murmurs caused by other intracranial vascular malformations, such as dural arteriovenous fistula and cerebral arteriovenous fistula, should also be excluded.

More than 90% of CCF patients can be successfully cured by intravascular interventional embolization [3, 12], which remains the first-line treatment for CCF. Here, the CCF on the right side of the patient was successfully cured by embolization using microcoil and Onyx liquid glue. The fistula on the left is comparatively large, due to which it is difficult to completely close the fistula with a detachable balloon. Therefore, multiple coils were introduced and finally the fistula was successfully embolized. The left CCF was embolized while the internal carotid artery was also occluded. Since the Willis circle is well compensated, even after the occlusion of the left internal carotid artery, the left cerebral hemisphere ischemia will not occur. Additionally, after occluding the left internal carotid artery, blood supply to the left ophthalmic artery is not completely possible, which may lead to progressive worsening or blindness in the left eye. Therefore, it is imperative that we should conduct longer follow-up of patients.

## Conclusions

Bilateral CCF secondary to craniocerebral injury does not always demonstrate the classic “triple syndrome,” which increases the difficulty of diagnose, and is often easily missed. Reporting and analyzing this case will help us elucidate the underlying mechanisms of this disease. We recommend that patients with skull base fractures should undergo a routine CTA examination to avoid missed diagnosis, to ensure that patients receive accurate and effective treatment.

## Data Availability

Not applicable
